# Unveiling the paradox—low ovarian reserve in ART pregnancies and the hidden opportunities in research

**DOI:** 10.1093/hropen/hoae008

**Published:** 2024-02-03

**Authors:** Liu Yang, Xingyu Sun

**Affiliations:** Clinical Medical College, Southwest Medical University, Luzhou, Sichuan, China; Department of Gynecology, The Affiliated Traditional Chinese Medicine Hospital, Southwest Medical University, Luzhou, Sichuan, China

Sir,

We write in response to the recent study by Chinè *et al.* (2023): ‘Low ovarian reserve and risk of miscarriage in pregnancies derived from assisted reproductive technology’. This study provides significant insights into the relationship between low ovarian reserve (as measured by anti-Müllerian hormone (AMH) and antral follicle count (AFC) levels) and miscarriage risk in ART pregnancies. However, we would like to highlight a few areas for further consideration and improvement.

First, while clinically relevant, the study’s focus on a younger demographic (under 35 years of age) potentially excludes insights into how age interplays with ovarian reserve in older women. Inclusion of a broader age range could provide a more comprehensive understanding of miscarriage risks across different age groups.

Second, the study’s retrospective design, while valuable, might limit the scope and precision of the data collected. Prospective studies could offer more detailed clinical information, potentially revealing new aspects of the relationship between ovarian reserve and miscarriage risk.

Additionally, it is worth noting that the study did not differentiate between women with spontaneous early exhaustion of ovarian reserve and those with anticipated exhaustion due to treatments like surgery or chemotherapy. Investigating these subgroups separately could yield insights into different pathogenetic mechanisms.

In a related systematic review and meta-analysis on the topic, Busnelli *et al.* (2021) identified a significant association between low AMH levels and increased miscarriage risk in ART pregnancies, highlighting the importance of serum AMH and AFC as markers in assessing ovarian reserve and reproductive outcomes. However, they also acknowledged the limitations in establishing a causal relationship due to study designs and recommended further well-designed studies to confirm these findings.

These considerations underscore the need for ongoing research to build upon the valuable findings of Chinè *et al.* (2023), enhancing our understanding of the complex dynamics of fertility, particularly in the context of ART.
